# Absence of the dermatan sulfate chain of decorin does not affect mouse development

**DOI:** 10.1186/s12952-017-0074-3

**Published:** 2017-04-17

**Authors:** Pierre Moffatt, Yeqing Geng, Lisa Lamplugh, Antonio Nanci, Peter J. Roughley

**Affiliations:** 1 Research Center, Shriners Hospitals for Children – Canada, 1003 Boulevard Décarie, Montréal, H4A 0A9 QC Canada; 2 Department of Human Genetics, Faculty of Medicine, McGill University, Montreal, QC Canada; 3 Laboratory for the Study of Calcified Tissues and Biomaterials, Department of Stomatology, Faculty of Dentistry, Université de Montréal, Montréal, QC Canada

**Keywords:** Decorin, Dermatan sulfate, Knockin mouse, Cartilage, Skin, Tendon, Cornea, Collagen, Development, Wound healing

## Abstract

**Background:**

In vitro studies suggest that the multiple functions of decorin are related to both its core protein and its dermatan sulfate chain. To determine the contribution of the dermatan sulfate chain to the functional properties of decorin in vivo, a mutant mouse whose decorin lacked a dermatan sulfate chain was generated.

**Results:**

Homozygous mice expressing only the decorin core protein developed and grew in a similar manner to wild type mice. In both embryonic and postnatal mice, all connective tissues studied, including cartilage, skin and cornea, appeared to be normal upon histological examination, and their collagen fibrils were of normal diameter and organization. In addition, abdominal skin wounds healed in an identical manner in the mutant and wild type mice.

**Conclusions:**

The absence of a dermatan sulfate chain on decorin does not appear to overtly influence its functional properties in vivo.

## Background

Decorin is a dermatan sulfate (DS) proteoglycan that belongs to the family of small leucine-rich repeat proteoglycans (SLRPs), which possess core proteins having central leucine-rich repeat regions flanked by disulfide-bonded domains and terminal extensions [1]. The decorin gene has 8 exons, with the protein sequence being encoded within exons 2–8 [2, 3]. The coding sequence possesses attachment sites for one DS chain within exon 2 and for one N-linked oligosaccharide within each of exons 5, 6 and 7. The disulfide-bonded domains are within exons 2 and 8.

The core protein of the mature form of decorin present in the extracellular matrix possesses a DS chain at amino acid residue 4 [4], although in different connective tissues the degree of epimerization of glucuronic acid to iduronic acid resulting in conversion of chondroitin sulfate (CS) to DS varies [5, 6]. The conversion of CS to DS may influence the properties of decorin because of differences in the ability of these glycosaminoglycans (GAGs) to self-associate and interact with proteins [7]. The decorin core protein can also be post-translationally modified with two or three N-linked oligosaccharides [8], but this difference does not appear to be of any functional consequence. Generation of the mature form of decorin not only involves removal of the signal peptide, but also an additional amino terminal peptide of 14 amino acids [9], which has been considered as a propeptide. It is likely that propeptide removal occurs via the action of bone morphogenetic protein 1 (BMP1), as this proteinase has been shown to cleave the propeptide from the structurally related SLRP, biglycan, at an amino acid sequence that is conserved in decorin [10]. At present it is not clear if the propeptide has a function on the secreted proteoglycan, but it does appear to play a role in intracellular trafficking [11].

Decorin has the ability to interact with collagen fibrils via amino acid sequences present within the leucine-rich repeats [12, 13]. Molecular modelling predicts that decorin possess a “horse-shoe” conformation that is able to accommodate a single collagen molecule at the surface of the collagen fibrils within its concave face [14, 15]. However, X-ray diffraction analysis of decorin crystals indicate that they exist as dimers with interlocking concave faces [16]. There is, however, some controversy over whether such dimers represent the functional form of the molecules in solution [17, 18], and how this impacts their interaction with the collagen fibrils.

Decorin has also been reported to interact with many other macromolecules, including structural molecules such as types VI, XII and XIV collagen, fibronectin and elastin [19–23], and growth factors such as EGF, TGFβ and TNFα [24–26]. These interactions may not only play a role in stabilizing the extracellular matrix, but also may participate in regulating its metabolism [27].

The importance of decorin in tissue function is best illustrated by the abnormal phenotypes arising in “knockout” mice. Absence of decorin results in lax, fragile skin, in which collagen fibril morphology is irregular with fusion of adjacent fibrils appearing to have occurred [28]. In the human, a frame shift mutation in the decorin gene gives rise to a congenital stromal dystrophy of the cornea [29]. The absence of DS synthesis can also have detrimental consequences on collagen architecture and tissue function, as deficiency in DS substitution of decorin due to mutation in a glycosyl transferase gene has been associated with the progeriod form of Ehlers-Danlos syndrome (EDS) [30, 31]. This mutation results in diminished DS substitution of decorin, but normal synthesis and secretion [31].

Thus it appears that both the decorin core protein and the DS chain may play a role in mediating its function. The purpose of the present study was to determine how absence of a DS chain on decorin due to mutation of the serine residue at the site of DS substitution influences development and growth of the skeleton and other connective tissues.

## Methods

### Generation of mouse decorin knockin (KI) genomic construct

**Fig. 1:**
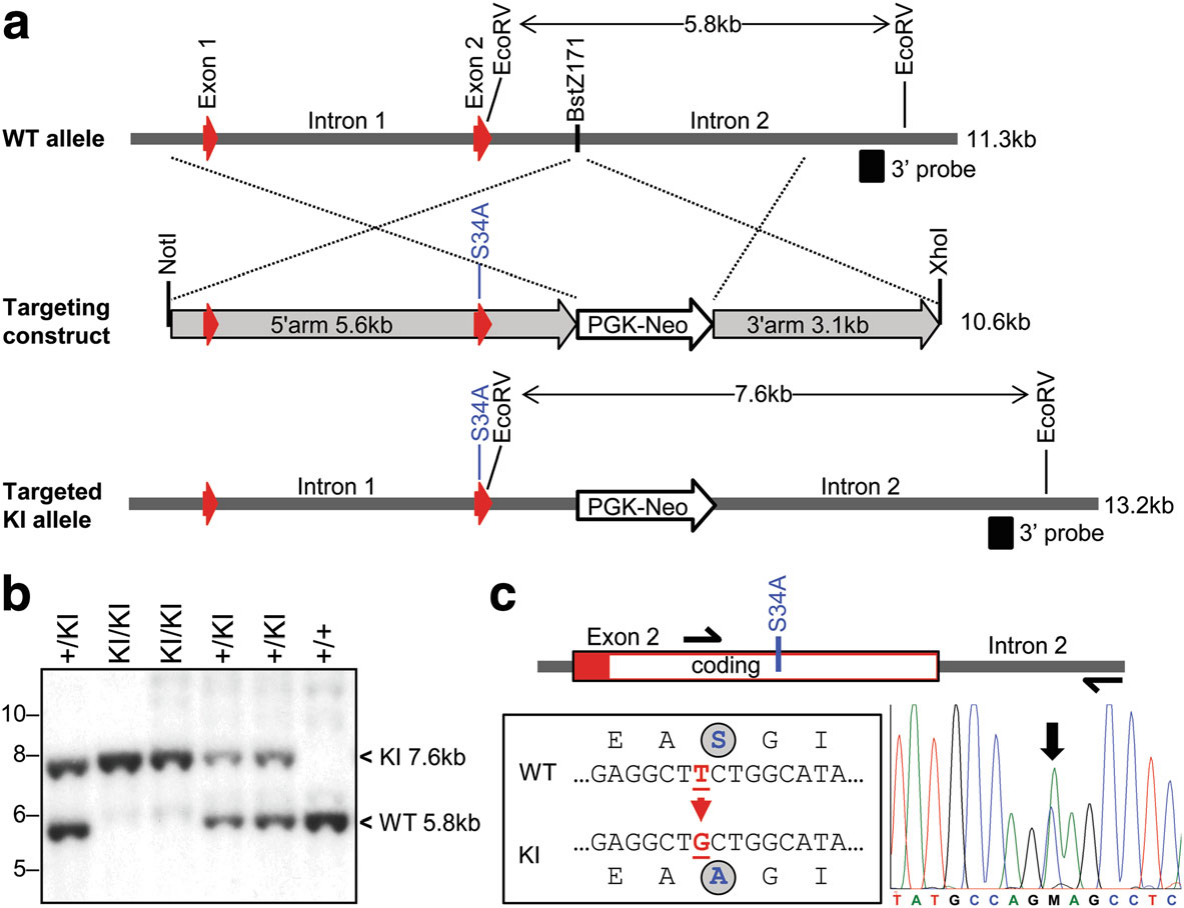
Strategy for generation of decorin KI mutant mice. **a** A targeting fragment (10.6 kb) spanning exon 1, intron 1, exon 2 and part of intron 2 was inserted into the murine decorin gene by homologous recombination. The targeting construct contained a PGK-Neo selection cassette inserted at the BstZ171 site, and a single nucleotide mutation within exon 2 of the serine codon at the DS attachment site for an alanine codon (see **c**). **b** Southern blotting of EcoRV-generated fragments from genomic DNA of wild type (+/+), heterozygous (+/KI) and homozygous (KI/KI) mutant mice. The 3’ probe used lies outside the targeting construct. **c** Nucleotide sequencing of the decorin gene in the region bearing the S34A knockin mutation in heterozygous mice. A 263 bp PCR fragment was amplified with the indicated primer locations (semi-arrows) and sequenced. The reverse complement chromatogram matches the sequence shown at left

BAC clone #228 L10 (Invitrogen) was used as a template to PCR amplify 5’arm (4469 bp) and 3’arm (4445 bp) gDNA fragments (Table 1, Fig. 1), which were then separately ligated into linearized pBluescript. The pBluescript-5’arm was used as a PCR template for mutagenesis using inverse PCR [32] with adjacent primers, one of which contained the GAG mutant site to change serine 34 to an alanine within exon 2 (Table 1). The linear PCR product was then religated using the overlapping EcoRV site within exon 2 to generate the pBluescript-5’arm containing the GAG mutant site. The pBluescript-3’arm was linearized with BstZ171, and a PGK-neomycin cassette was inserted. The modified 5’arm and 3’arm were excised with EcoRV and XhoI to generate fragments of 6262 bp and 7281 bp, which were ligated to make pBluescript-KI. HindIII or BamHI cleavage was used to select the correct clones. The identity of the final constructs was confirmed by Sanger sequencing on an Applied Biosystem 3730xl DNA Analyzer through the McGill University and Genome Quebec Innovation Centre. The KI fragment (10.6 kb) was excised from the remaining vector sequence using XhoI and NotI. The linear KI fragment (1.3 μg/μl) was supplied to the Goodman Cancer Center Transgenic core facility at McGill University for electroporation into 129sv R1 ES cells.

**Table 1:** Oligonucleotide primers used for generation of DS-deficient decorin KI mouse

Purpose	Oligo name	Sequence (5’ > 3’)
Generation of arms for mouse decorin homologous recombination		
	5’arm_FWD	CACTTGAACTTCAGAGCCCAGGA
	5’arm_REV	GGCCAAGCACTCAAGAGAACTC
	3’arm_FWD	GGGCTGGACCATTTGAACAGA
	3’arm_REV	CCTTGGAGGTACGAATCATTGTG
Mutagenesis of 5’ arm to introduce the S34A change (underlined base)		
	5’arm_S34A_FWD	CTGGCATAATCCCTTATGACC
	5’arm_S34A_REV	CAGCCTCATCTTCTAGCATGAAG
3’ genomic probe for Southern hybridization		
	Probe_FWD	CCAAGGTATTTCCAACTCAGTG
	Probe_REV	CATGTGTGCCTAGTGATGATGG
PCR genotyping and sequencing		
	Genotyping_FWD	GGGCTGGACCATTTGAACAGA
	Genotyping_REV	GGGTGTGTCTCTTACTTCTGGA

Five hundred neomycin resistant ES cell clones were screened for recombination and integration of the KI decorin allele as described [33]. Cells in 96-well plates were treated with proteinase K (0.5 mg/ml in 10 mM Tris–HCl (pH 7.5), 10 mM EDTA, 0.5% (w/v) Sarkosyl) and incubated at 55 °C overnight. The gDNA was precipitated with 75 mM NaCl in 100% ethanol, washed with 70% ETOH, then digested with EcoRV. The EcoRV-digested gDNA was analyzed by 1% agarose gel electrophoresis, transferred to positively charged nylon membrane, and probed by Southern blot with a [α-^32^P] dCTP (3000 Ci/mmol) (PerkinElmer Life Sciences) random primed-labeled decorin 3’ probe or a neomycin probe. Positive ES clones were selected, expanded in 6-well plates, and reanalyzed to confirm their identity. Positive mutant ES cell clones were injected into separate blastocysts by the Transgenic core facility, then implanted into pseudopregant females to generate chimeric mice.

### Generation of mutant mice

Chimeric mutant mice were crossed with C57Bl6 wild-type mice to generate heterozygote mutant mice. Heterozygous males and females were then bred to each other to generate homozygous KI mice. Homozygous mice were bred to one another to propagate the homozygous line.

### Analysis of phenotype of mutant KI mice

#### Histology

Postnatal mice were euthanized by CO_2_ asphyxia and dissected for cornea, limb and tendon samples. Tissues (or embryos) were fixed in 4% paraformaldehyde/PBS for 48 h at 4 °C. Femur length of 2 month old mice (5 of each genotype) was evaluated using a fine digital caliper. Bone samples were decalcified at 4 °C in 10% EDTA/0.1 M Tris–HCl, pH 7.4 and processed for paraffin embedding using standard methods. Six μm thick paraffin sections were cut and stained with Alcian blue/nuclear fast red. Sections were mounted with microkitt and photographed with a Leica DMRB microscope equipped with an Olympus DP70 camera. Growth plate height measurements were derived from pictures of selected sections showing comparable histological architecture. The total growth plate height was averaged from measurements done at 5 different sites along the width of the bone on 5 individual mouse femurs of each genotype.

#### Skeletal preps

E18.5 staged pregnant females were euthanized by CO_2_ asphyxia and embryos dissected into cold PBS. Embryos were skinned, eviscerated and fixed in 95% ethanol for 24 h, then incubated in 100% acetone for 24 h. Embryos were stained in Alcian blue/alizarin red for 6 h at 37 °C and then overnight at room temperature. Embryos were rinsed briefly in distilled water and transferred into 1% aqueous potassium hydroxide for 2 h. The embryos were transferred to 1% KOH/20% glycerol until cleared and then transferred progressively through a glycerol gradient (50%, 80% and 100%) over 2 days.

### Electron microscopy of mouse tissues

The eyes and hind limbs were dissected from 2 month old animals. Samples were fixed in 5% glutaraldehyde in 0.1 M sodium cacodylate buffer, pH 7.3 for 48 h. For the first 24 h, Achilles tendons were left attached to the folded hindlimbs to maintain extension. Tendons were subsequently dissected for the final 24 h of fixation. Tissues were post-fixed in 1% osmium tetroxide/1.5% potassium ferrocyanide for 2 h on ice, dehydrated through an increasing concentration of acetone, and infiltrated and embedded in Epon resin. Preparation of ultrathin sections and imaging were essentially as described previously [34]. Low magnification micrographs from wild type and knockin mice (*n* = 3) were visually inspected to have comparable tissue architecture. For the Achilles tendons, high power (21,000×) magnifications of corresponding fields were obtained and fibril diameters counted manually using an eyepiece graticule.

### Isolation of decorin from cartilage and fibroblasts

Cartilage was used for direct matrix protein extraction. Costochondral cartilage was dissected from day 5 postnatal mice, cleaned off of muscles and connective tissues, cut into small tissue pieces, and snap frozen in liquid N_2_. Frozen samples were then ground to a fine powder with a mortar and a pestle and transferred to an Eppendorf tube containing 0.5 ml of 100 mM Tris-acetate (pH 7.5) containing a protease inhibitors cocktail (Sigma P8340). The resuspended material was split into 2 equal halves, 0.06 unit of *P. vulgaris* chondroitinase ABC (Seikagaku) was added to one and the other was left untreated. After being incubated at 37 °C for 16 h with gentle mixing, cartilage was spun down at 10000 *g* for 5 min and boiled with reducing Laemmli sample buffer at 100 °C for 3 min. Samples were analyzed by 10% SDS-PAGE and western blotting using the polyclonal anti-decorin antiserum LF-113 kindly provided by Dr. Larry Fisher (NIH) [35]. Briefly, proteins were transferred to nitrocellulose by electroblotting and the membranes were monitored by Ponceau red staining to ensure uniform transfer. Membranes were blocked for 1 h with 5% skim milk PBS containing 0.05% Tween (PBS-T), and incubated overnight at 4 °C with the anti-decorin antiserum diluted 1:1000 in blocking solution. After washing with PBS-T, blots were incubated for 1 h at room temperature with an anti-rabbit-HRP-coupled antibody (Amersham) diluted 1:30000 in blocking solution. Immunoreactive proteins were visualized using an enhanced chemiluminescent ECLprime detection reagent (Amersham Biosciences) and exposure to hyperfilm.

Skin was used to study decorin production by fibroblasts. Abdominal skin (1 × 1.5 cm) from day 5 postnatal mice was collected in PBS, cut into small pieces, then digested with 0.2% collagenase D (Roche) in 15 ml DMEM with penicillin at 37 °C for 3 h. Fibroblasts were recovered by filtration through a cell strainer and subsequent centrifugation at 1000 *g* for 10 min. Cells were suspended in 6 ml DMEM containing 10% FBS in a P60 Petri dish and incubated at 37 °C/5% CO_2_. At day 3, 2/3 of the medium was replaced with fresh medium, and at day 5 cells were passaged at a ratio of 1/5. When cells reached confluency, they were rinsed 3-times and incubated with serum-free DMEM for 24 h. The spent media was collected and centrifuged at 10000 *g* for 5 min to remove floating cells and debris. Proteins in the media were precipitated with 10% (v/v) trichloroacetic acid for 30 min on ice and centrifuged. The protein pellet was washed once with cold acetone, dried, and resuspended with a buffer containing 50 mM Tris–HCl (pH 7.5), 150 mM NaCl, 1 mM EDTA, 1% NP40, and protease inhibitors. Laemmli sample buffer was added and the samples were processed for SDS-PAGE and western blotting analysis as described above.

### Wound healing of mouse skin

An excisional skin wound healing procedure was performed as described [36]. In brief, 2 month old mice were anaesthetized with isoflurane and hair removed with a razor between the shoulder blades. Two dorsal skin punches were removed using a disposable 4 mm skin biopsy punch (Acuderm Inc., Ft. Lauderdale, FL), and animals were treated with a subcutaneous injection of carprofen analgesic then allowed to regain consciousness in a heated environment. Skin punches were photographed at various time points to chronicle healing. At each time point, animals were euthanized, and skin was removed from around the healing punch site and placed dermis side down on a piece of Whatman filter paper to maintain flatness. Tissue was fixed in PLP solution [37] overnight at 4 °C, rinsed in PBS and processed for either paraffin embedding or cryo-embedding. Skin samples were sectioned, stained with hematoxylin/eosin and photographed as previously described. The wound healing experiment was performed twice on groups of 3 mice per genotype each time.

## Results

**Fig. 2:**
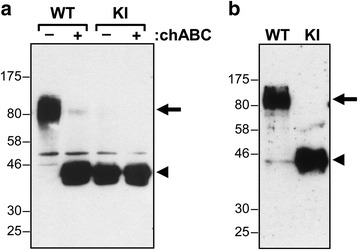
Western blotting of decorin from day 5 postnatal wild type (WT) and homozygous knockin (KI) mutant mice. **a** Decorin directly extracted from costochondral rib cartilage. **b** Decorin secreted by skin fibroblasts. Samples were either analyzed directly (−) or following treatment with chondroitinase ABC (+), and the position of intact decorin is indicated by an arrow and DS-deficient decorin by an arrowhead. Migration position of molecular mass (kDa) markers is indicated at left

Homozygous KI mice bearing a serine to alanine substitution at the DS attachment site of decorin were generated (Fig. 1). These mice should differ from WT mice only in their inability to produce the proteoglycan form of decorin. Instead they should produce only the decorin core protein with its N-linked oligosaccharides. To verify that the mutant mice did indeed produce only decorin missing its DS chain, the structure of the decorin was analyzed by SDS/PAGE and immunoblotting following direct extraction of rib cartilage or following its production by skin fibroblasts. Analysis of the rib cartilage showed only the proteoglycan form of decorin in the WT mice and only the decorin core protein in the homozygous mutant mice (Fig. 2a). The cartilage from the heterozygous mutant mice possessed similar amounts of both forms of decorin (Fig. 2a). Analysis of the decorin secreted into the culture medium by skin fibroblasts showed only the proteoglycan form of decorin being produced by WT cells and only the decorin core protein being produced by homozygous KI cells (Fig. 2b). Thus the tissues of the homozygous mutant mice produce only the core protein of decorin, which appears to be present at a similar abundance to the proteoglycan form of decorin in the WT mice.

**Fig. 3:**
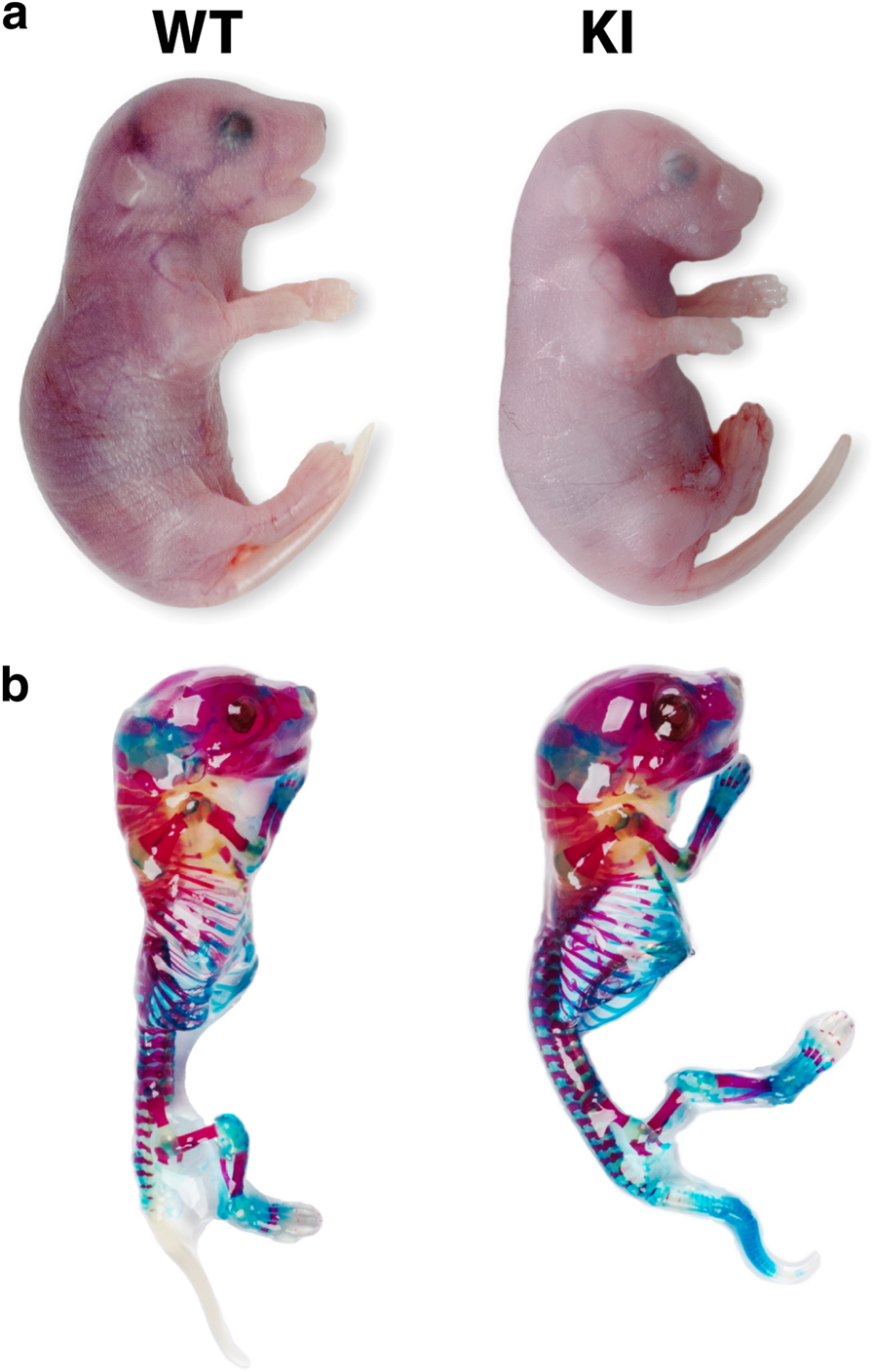
Gross appearance and skeletal preparations of embryonic wild type (WT) and knockin (KI) mutant mice. **a** Appearance of E18.5 embryonic mice. **b** Skeletal preparations of E18.5 mice stained with Alcian blue and Alizarin red

**Fig. 4:**
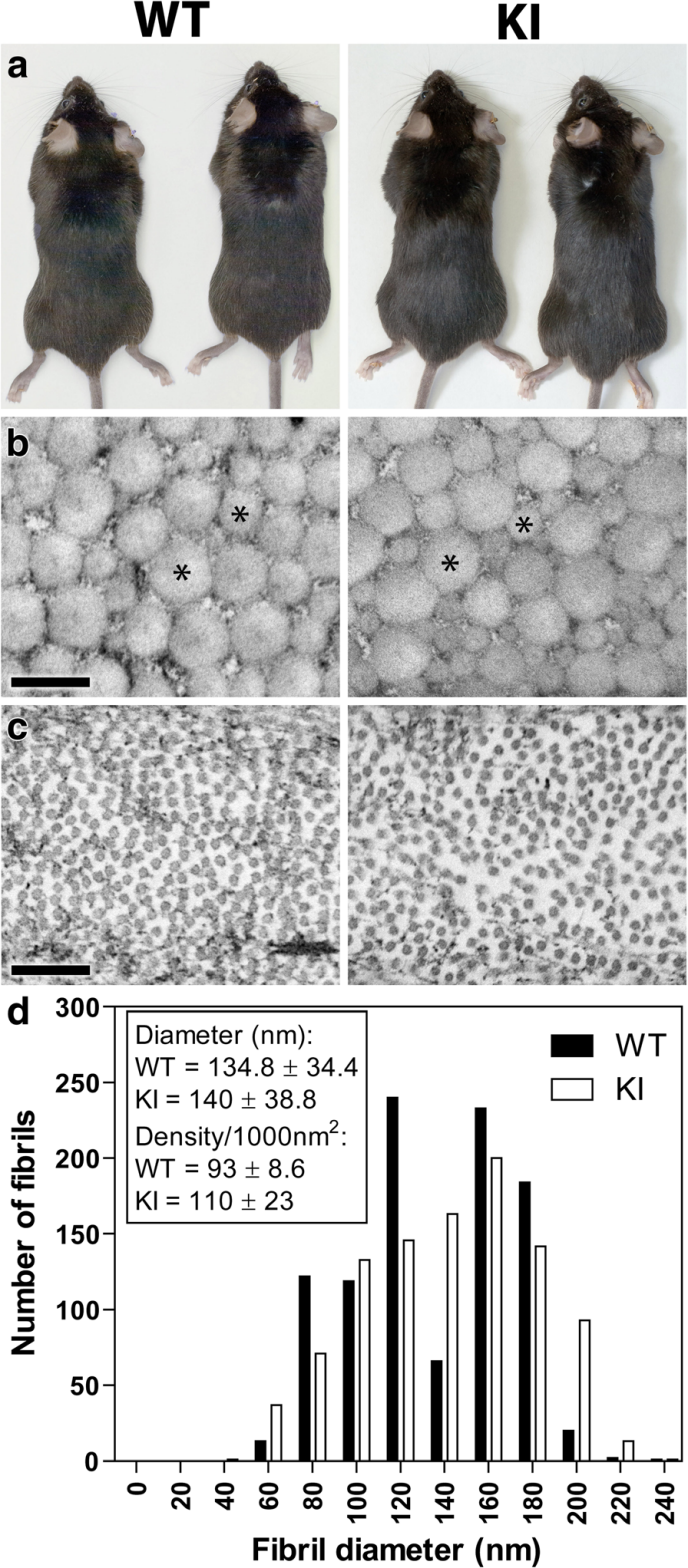
Gross appearance and transmission electron microscopy (TEM) of 2 month-old postnatal wild type (WT) and knockin (KI) mutant mice. There is no significant difference in appearance of mice (**a**) and in the cross-section profiles of collagen fibrils of the tendon (**b**) and cornea (**c**). Asterisks label 2 collagen fibers in each panel. **d** Quantification of Achilles tendon fibril distribution as a function of diameter size shows no major changes between genotypes (black bars WT; open bars KI). The inset text box reports the mean fibril size and density. Scale bars = 250 nm

**Fig. 5:**
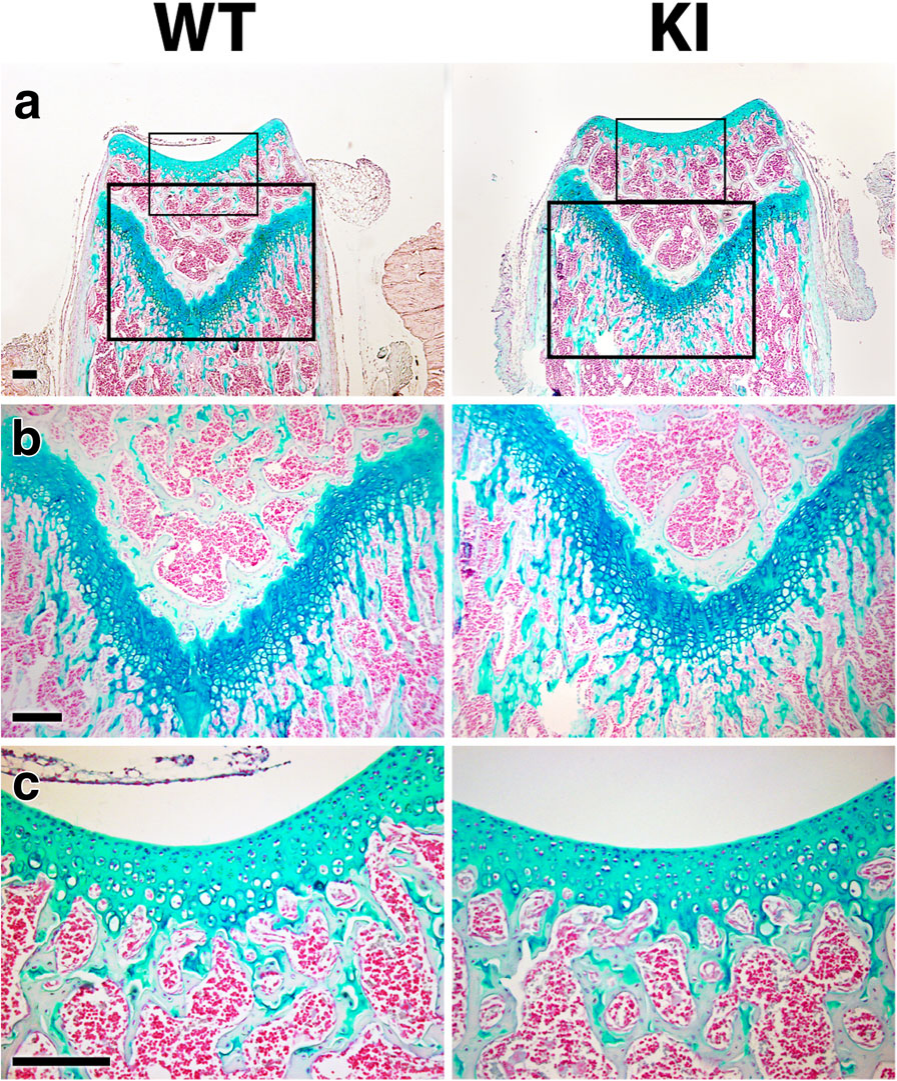
Histology of cartilage from postnatal wild type (WT) and knockin (KI) mutant mice. **a** Histology of distal femur of 2 month old mice stained with Alcian blue. **b** Higher magnification of the growth plate. **c** Higher magnification of articular cartilage. Sections were counter stained with nuclear fast red. Scale bars = 0.15 mm

Both the embryonic and postnatal KI mice showed no obvious difference in gross appearance from WT mice (Figs. 3a and 4a), with body size and limb and tail lengths being similar. Analysis of the whole skeleton in E18.5 embryonic mice showed no apparent difference in bone size or shape throughout the axial and appendicular skeletons and the skull (Fig. 3b). Whole body weights recorded for 2-month old male mice did not differ significantly (25.7 g ± 0.4 versus 24.6 g ± 2.6 (average ± SD (*n* = 3)) for WT and KI mice, respectively). The collagen fibril appearance and structure in both the cornea and Achilles tendons of 2 month old mice also showed no clear difference between the KI and WT mice (Fig 4b and c). Quantitative measurements of Achilles tendon collagen fibril density, diameter and distribution did not reveal any significant changes (Fig. 4d). In addition, there was no observable difference in the structure, thickness and cellular organization of the articular cartilage or growth plate in the femur of the 2 month old mice (Fig. 5a-c). On average, the height of the growth plate measured across the entire width of the distal femur was similar in both genotypes (182 μm ± 42 versus 183 μm ± 32 for the WT and KI mice, respectively (average ± SD (*n* = 5)). Consistently, the total femur length was also not significantly different between WT and KI mice at 2 months of age (15.7 mm ± 0.3 versus 16.1 mm ± 0.3, respectively (average ± SD (n = 6)). Thus it would appear that the absence of the DS chain on decorin does not impair development or growth of the mutant mice.

**Fig. 6:**
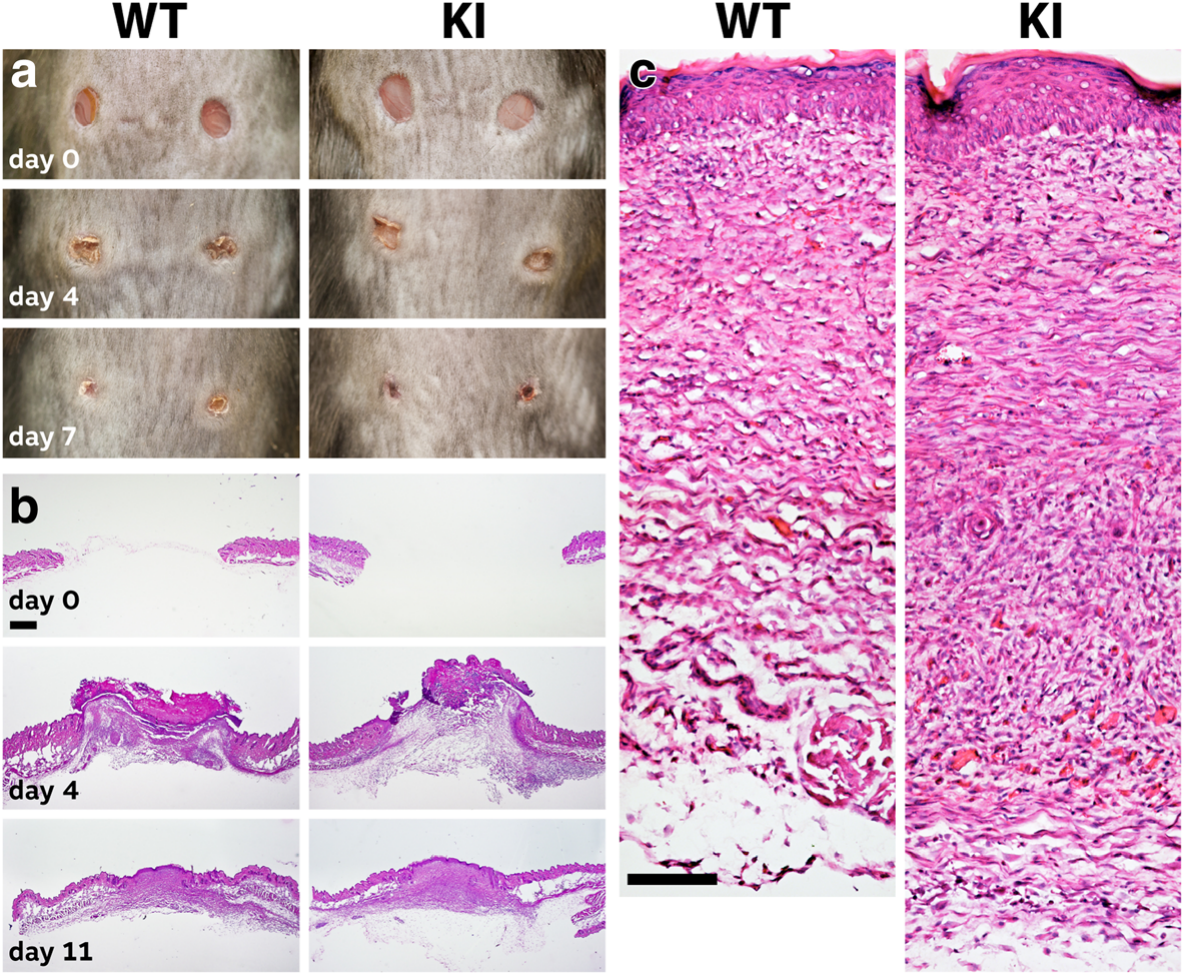
Wound healing in 2 month old wild type (WT) and knockin (KI) mutant mice. **a** Appearance of wounds at the time of injury and after 4 and 7 days of healing. **b** Histology of wound healing site stained with hematoxylin/eosin at time of wounding and at days 4 and 11 after wounding. Scale bar = 1 mm. **c** Higher magnification of dermis after 11 days of wound healing. Scale bar = 0.2 mm

Finally, to determine whether absence of the DS chain influences a pathological process in which decorin is thought to participate, wound healing of the skin was studied. In the 2 month old mice studied, circular punch wounds in the dorsal skin healed at the same rate in both WT and homozygous KI mice (Fig. 6a and b). Histological analysis also revealed that the organization of the repaired skin appear the same in both the WT and KI mice (Fig. 6c). Thus there was no evidence that the absence of the DS chain on decorin had any impact on wound healing.

## Discussion

The absence of any major phenotypic change in mice lacking a DS chain on decorin is somewhat surprising based on reports in the literature, which suggests that both components of the decorin molecule play a functional role [38]. Both the decorin core protein and its DS chain have been implicated in the interaction with TGFβ, TNFα, FGF2 and FGF7 [24, 26, 39–41]. As these growth factors and cytokines play important roles in both physiology and pathology, one might predict that mice lacking the DS chain on decorin would show abnormal traits. Yet such abnormality was not apparent.

It has been reported that absence of the DS chain on decorin influences the mechanical properties of newly formed cartilage generated in vitro, suggesting that the DS chain is important in the organization/maturation of cartilage [42]. Hence, one might have expected a perturbation in skeletal development in the KI mice, but this did not occur. However, this lack of a phenotypic change is not entirely unexpected as there is little evidence for altered cartilage integrity in the decorin knockout mouse, though these mice do show differences in the compressive stiffness of their articular cartilage [43]. The DS chain of decorin has also been reported to play a role in wound healing, both by influencing collagen formation during the early stages of fibrillogenesis [44] and through impairment of fibroblast function via modulation of the α2β1 integrin and vimentin intermediate filament systems [45]. However, the KI mice exhibited no delay in wound healing of skin lesions, and there was no variation in collagen fibril diameter between WT and KI mice.

Defects in the DS chain of decorin have also been implicated in some human disorders [46]. Impaired DS substitution of decorin has been associated with some rare forms of EDS, involving mutations in either the galactosyl transferase gene that participates in formation of the linkage region between DS and the protein to which it is attached [47, 48] or the sulfotransferase gene involved in DS sulfation [49]. As with other forms of EDS, the affected patients exhibit abnormalities in collagen formation. Again this suggests that absence of the DS chain on decorin should influence connective tissue formation.

This apparent discrepancy between previous work and the present report can be reconciled if one postulates that both DS and the decorin core protein are essential for normal tissue function, but that the DS does not necessarily have to be attached to the decorin core protein. In the KI mice used in the present work there is no DS on the decorin but DS will be present in the extracellular matrix on other proteoglycans such as biglycan. It is possible that such DS can compensate for the function of that normally attached to decorin, and that a phenotype will only result if all DS is affected, such as in the Ehlers-Danlos cases. Thus while the DS chain on decorin may be essential for normal function in in vitro experiments where it is the only or major source of DS, it may not be essential in vivo where DS compensation can occur.

It is also possible that the function of the DS chain on decorin is not conserved between all species or even tissues, as it is known that the degree of epimerization of DS can vary with both of these parameters [50] and that the interactions of DS can be dependent on its structure. Thus it is possible that the structure of the DS on mouse decorin is such that it does not participate in decorin function. As such its loss would not then be expected to alter decorin function in this species. If one accepts this premise then it is possible that in other species, such as the human, the absence of DS on decorin could be detrimental. In the ExAC database [51], which contains the genomic DNA sequence of over 60,000 individuals, no single nucleotide polymorphisms exist in the serine 34 codon of human decorin. Thus it is not possible to predict the clinical outcome if a mutation preventing DS substitution in humans would occur and whether it would cause any significant phenotype or behave in a similar manner to the mouse.

## Conclusions

The absence of a DS chain on decorin does not appear to overtly influence its functional properties in vivo during tissue development and growth or during wound healing in the skin.

## Abbreviations

CS: Chondroitin sulfate
DS: Dermatan sulfate
EDS: Ehlers-Danlos syndrome
GAG: Glycosaminoglycan
KI: Knockin
SLRPs: Small leucine-rich repeat proteoglycans
WT: Wild type
